# CmirC update 2024: a multi-omics database for clustered miRNAs

**DOI:** 10.1007/s10142-024-01410-2

**Published:** 2024-08-01

**Authors:** Akshay Pramod Ware, Kapaettu Satyamoorthy, Bobby Paul

**Affiliations:** 1 Department of Bioinformatics, Manipal School of Life Sciences, Manipal Academy of Higher Education, Manipal, Karnataka 576104 India; 2 Institute of Cardiovascular Regeneration, Johann Wolfgang Goethe University, Theodor-Stern-Kai 7, Frankfurt Am Main, 60590 Germany; 3 SDM College of Medical Sciences and Hospital, Shri Dharmasthala Manjunatheshwara (SDM) University, Manjushree Nagar, Sattur, Dharwad, Karnataka 580009 India

**Keywords:** DNA methylation, Cancer informatics, Integrated data analysis, Epigenomics

## Abstract

Clustered miRNAs consist of two or more miRNAs transcribed together and may coordinately regulate gene expression. Differential expression of clustered miRNAs is found to be controlled by crosstalk of genetic or epigenetic mechanisms. It has been demonstrated that clustered miRNA expression patterns greatly impact cancer cell progression. With the *CmirC* initiative, we initially developed a comprehensive database to identify copy number variation (CNV) driven clustered miRNAs in cancer. Now, we extended the analysis and identified three miRNAs, mir-96, mir-183, and mir-21, were found to be significantly upregulated in 17 cancer types. Further, *CmirC* is now upgraded to determine the impact of changes in the DNA methylation status at clustered miRNAs by utilizing The Cancer Genomic Atlas (TCGA) cancer datasets. We examined specific methylation datasets from 9,639 samples, pinpointing 215,435 methylation sites and 27,949 CpG islands with miRNA cluster information. The integrated analysis identified 34 clusters exhibiting differentially methylated CpG sites across 14 cancer types. Furthermore, we determined that CpG islands in the promoter region of 20 miRNA clusters could play a regulatory role. Along with ensuring a straightforward and convenient user experience, *CmirC* has been updated with improved data browsing and analysis functionalities, as well as enabled hyperlinks to literature and miR-cancer databases. The enhanced version of *CmirC* is anticipated to play an important role in providing information on the regulation of clustered miRNA expression, and their targeted oncogenes and tumor suppressors. The newly updated version of *CmirC* is available at https://slsdb.manipal.edu/cmirclust/.

**Supplementary Information:**

The online version contains supplementary material available at 10.1007/s10142-024-01410-2.

## Introduction

The discovery of microRNAs (miRNAs) in the early 1990s revealed an unanticipated level of gene expression control that has been shown to be of enormous importance in the regulation of several physiological and pathological states, including carcinogenesis, cancer progression, and response to treatment (Condrat et al. 2020). Furthermore, growing evidence supports miRNA’s potential involvement as disease-specific biomarkers, resulting in an intriguing new tool for diagnostic, preventative, or therapeutic purposes (Zhang et al. 2022). The importance of miRNA-mediated regulation is emphasised even further by the fact that miRNA genes exhibit a tendency to cluster across the genome, and this clustering is substantially conserved across species (Altuvia et al. 2005; Wang et al. 2016). Each cluster comprises two or more miRNAs that are transcribed from adjacent genomic locations through a sole promoter region (Seitz et al. 2004). According to the miRBase repository, more than 40% of the experimentally confirmed human miRNA cluster genes have been found inside the 10 kb area (Kozomara et al. 2019). Members of clusters typically exhibit significant sequence similarity in the seed region and often target genes within the same pathway, either collectively or individually. Consequently, the impact of abnormal expression of clustered miRNAs might be more pronounced compared to non-clustered ones. Together, these clusters have the potential to regulate various facets of cellular activity, such as growth, proliferation, infection, signalling, metabolism, differentiation, development, cell death, immunity, organelle formation, DNA repair, and self-renewal. The dysregulation of miRNA clusters, resulting in changes to biological functions, plays a pivotal role in the development of numerous diseases, including cancer (Ware et al. 2022a).

In cancer development, alterations in clustered miRNA expression can occur through genetic mutations, deletions, amplifications, and DNA methylation (Gregorova et al. 2021). These, in turn, can have a significant impact on targeted genes and signal transduction pathways. Hence, we performed an integrated multi-omics analysis and in June 2022, we launched the first version of *CmirC*, a database of copy number variations (CNVs) colocalized clustered miRNAs in 35 TCGA cancer types (Ware et al. 2022b). The primary goal of this database was to decipher the detailed information on CNV-driven clustered miRNAs in cancer types. Further, we sought to get a clearer picture and a comprehensive understanding of DNA methylation mediated clustered miRNA regulation in cancer. With this in view, we have upgraded the *CmirC* web portal, and the current version includes DNA methylation datasets belonging to 14 TCGA cancer types. To enhance the *CmirC* 2022, we have (i) retrieved DNA methylation datasets; (ii) performed an integrative multi-omics analysis to identify the cancer-specific differentially expressed miRNAs; (iii) identified the internal regulators of clustered miRNAs; and (iv) upgraded the *CmirC* web-portal for multi-omics interactive analysis. The new version of this web portal is now hyperlinked with other independent databases and portals to obtain additional information. Through rigorous testing, we have identified and minimized multiple bugs, updated the database, and made improvements to enhance the user experience.

## Materials and methods

**Fig. 1:**
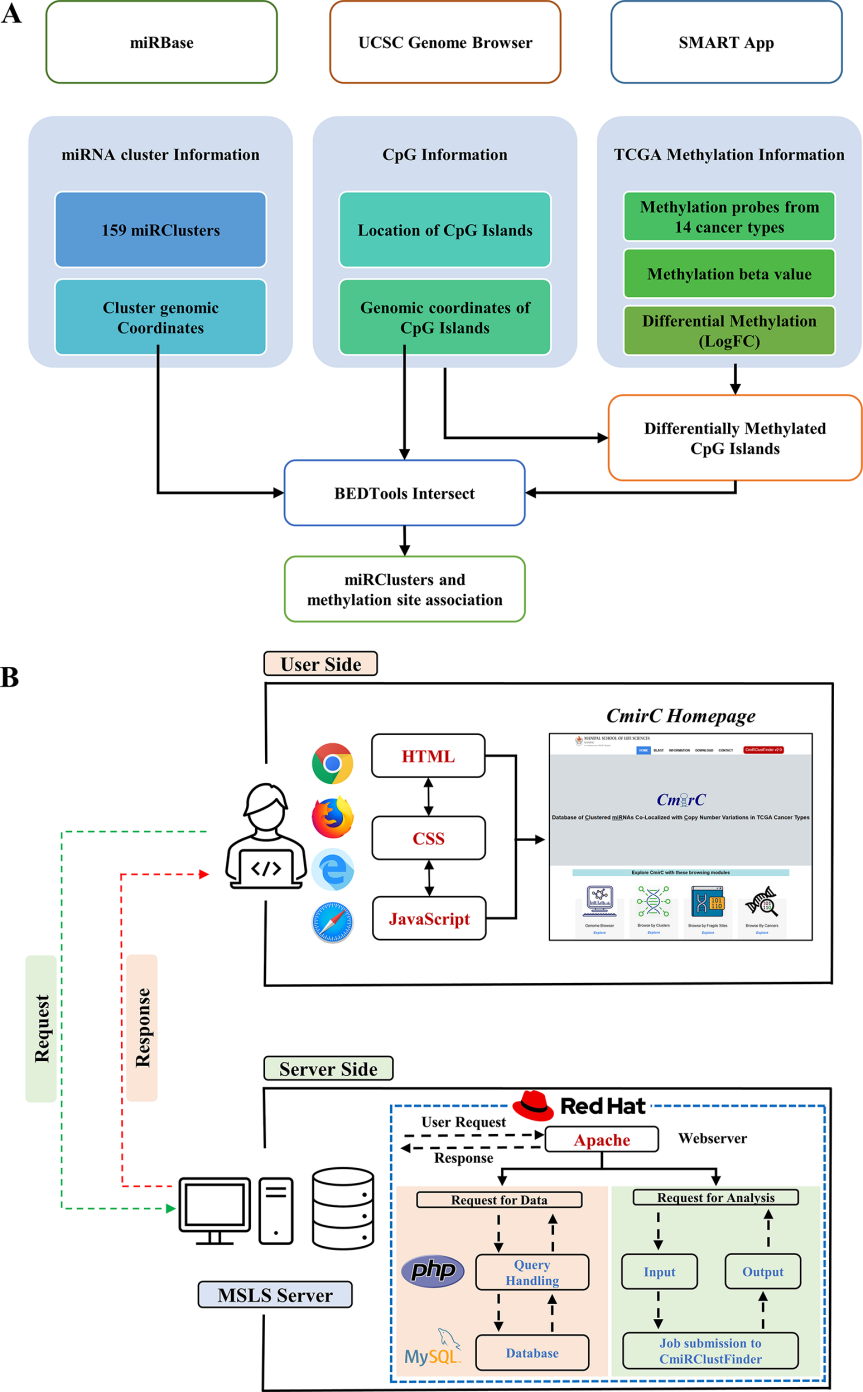
(**A**) Schematic representation of data collection, analysis and integration. (**B**) Overall working process of *CmirC* web portal. Entire data referenced against the human reference genome hg38

The revamped *CmirC* portal is powered by a variety of programming and scripting languages, including HTML, PHP, Bash Script, JavaScript, and MySQL. Figure 1 provides a visual depiction of the data collection, analysis, integration processes, and overall functionality of the web portal.

### Integrated multi-omics data analysis

#### Data acquisition and sources

The level 3 miRNA expression datasets, recurrent copy number variation (RCNV) details, miRNA cluster information and their coordinates were retrieved from the TCGA-GDC (https://portal.gdc.cancer.gov/) and the *CmirC* (Ware et al. 2022b) web portals. A list of all TCGA cancer datasets, cancer types, and sample sizes used in the study can be found in Supplementary Table 1. The table browser functionality of the UCSC genome browser’s (Kent et al. 2002) was used to download all of the CpG island maps in the human genome. The information on differentially methylated (DM) CpG sites/probes across 14 TCGA cancer types was obtained through the SMART App (Li et al. 2019). Table 1 presents the specific numbers of DM probes obtained for each cancer type.

**Table 1:** Distribution of differently methylated probes across 14 TCGA cancer types utilized for the upgradation of the CmirC

TCGA Abbreviation	Cancer	Total no of DM probes
BLCA	Bladder Urothelial Carcinoma	29,256
BRCA	Breast invasive carcinoma	12,962
COAD	Colon adenocarcinoma	23,753
ESCA	Esophageal carcinoma	11,339
HNSC	Head and Neck squamous cell carcinoma	14,963
KIRC	Kidney renal clear cell carcinoma	5050
KIRP	Kidney renal papillary cell carcinoma	6510
LIHC	Liver hepatocellular carcinoma	31,339
LUAD	Lung adenocarcinoma	5334
LUSC	Lung squamous cell carcinoma	19,345
PAAD	Pancreatic adenocarcinoma	4007
PRAD	Prostate adenocarcinoma	12,146
THCA	Thyroid carcinoma	1968
UCEC	Uterine Corpus Endometrial Carcinoma	37,463

#### Data processing

In the SMART App tool, hypermethylation and hypomethylation sites were selected using a beta-value cutoff of > 0.25 and an adjusted p-value of < 0.05. We performed differential gene expression analysis ( cancer cells vs. normal cells) using the TCGAanalyze_DEA function from the Bioconductor edgeR package (Robinson et al. 2010). The pair-wise tests were conducted using the ‘glmLRT’ function to compare the two groups. The obtained adjusted p-values were sorted in ascending order and subsequently subjected to identify the top differentially expressed miRNAs (DEmiRs). Consequently, DEmiRs were deemed significant only if logarithmic fold change (Log2FC) > 1.5 and adjusted p value < 0.05.

#### Integrated data analysis

BEDTools (Quinlan and Hall 2010) were utilized to overlap the genomic coordinates of significant RCNV, differentially methylated CpG probes, and CpG islands onto the clustered miRNAs. We have identified the clustered miRNAs on RCNV regions that are only partially colocalized, along with the methylation sites found both upstream (up to 20 kb) and internally within the clusters. The hg38 genome build was utilized in the study, and the UCSC LiftOver (https://genome.ucsc.edu/cgi-bin/hgLiftOver) tool was used to uplift the genomic coordinates between assemblies as needed. We performed the Kaplan–Meier plot analysis within the miRpower tool (Lánczky et al. 2016) to assess relapse-free survival (RFS) with default clinical parameters. The miRNA target genes were obtained from the miRTarbase to map upregulated oncogenes and downregulated tumor suppressors.

#### Statistical analyses and data visualization

In this study, R studio (version 4.1.2) was utilized to conduct all statistical analyses. The visualization of DEmiRs and the top 20 frequently expressed miRNAs across 19 cancer types was accomplished using the R Bioconductor package ggplot2 (Wickham 2016). Illustrations of mapped CpG islands and DM probes were performed using the Circos tool (Krzywinski et al. 2009 ). A significance level of adjusted p value < 0.05 was applied throughout the study.

### *CmirC* upgradation

#### Reconfiguration of genome browser

We have added DNA methylation tracks to the genome browser powered by JBrowse (Skinner et al. 2009) in the *CmirC* while preserving the existing tracks. GFF3sort (Zhu et al. 2017) a Perl based program was used to convert the BED files with DM probes retrieved from the SMART App to GFF3 files. Furthermore, JBrowse requires GFF3 in GFF3Tabix format, which we converted using GenomeTools (Gremme et al. 2013). The Multi-check box menu and separate track have been provided to access DM probes across 14 TCGA cancer types. Also, the annotated CpG islands in the human genome were provided in a separate track.

#### Interactive resources hyperlinked to *CmirC*

We hyperlinked all the clustered miRNAs to UALCAN (Chandrashekar et al. 2022), an integrated cancer data analysis platform that allows for Kaplan-Meier (KM) analyses. Genes and clustered miRNAs are also linked to various additional resources, including expression data (miRcancer, GEO), cancer associations (dbDEMC, miRNASNP, OMCD), literature sources (PubMed, Scopus, ScienceDirect), and miRNA targets (TargetScan, miRWalk). These links assist users in exploring the diverse features available in these databases. The data in the upgraded CmirC can be downloaded in text, GFF3, PNG, and BED file formats. These data files can be directly provided to other web-based or standalone tools for further analysis.

#### Bugs minimization

*CmirC* runs on LAMP, a stack of open-source software based on Linux. The components of LAMP include Apache v2.4, MySQL v5.7, and PHP v7.2, and *CmirC* is supported by HTML, cascading style sheets, JavaScript, bootstrap, PHP, and MySQL. Upon the initial release of *CmirC*, we conducted comprehensive testing of the web portal. We invited both internal and external users to explore and provide feedback on their user experience with *CmirC*. We also conducted extensive debugging of various scripts at multiple levels wherever necessary. To sum up, these improvements make *CmirC* one of the most research-friendly databases for clustered miRNAs, providing extensive multi-omics data.

## Results and Discussion

### Identification of DEmiRs in TCGA cancers

**Fig. 2:**
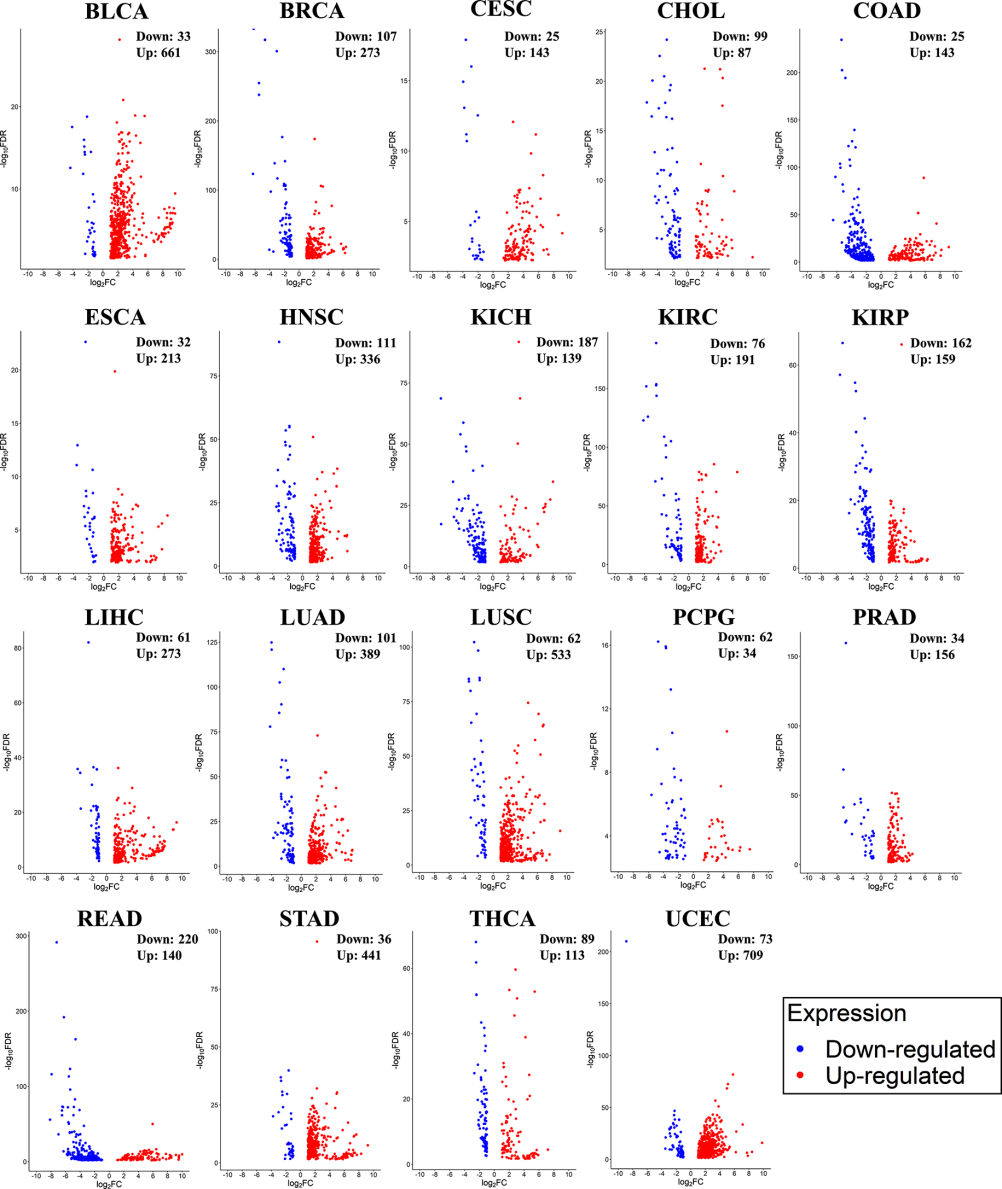
Cancer specific differentially expressed miRNA ( cancer cells vs. normal cells) analysis utilizing TCGA miRNA expression datasets. Log2FC above 1.5 considered as upregulation and below − 1.5 considered as downregulation

**Fig. 3:**
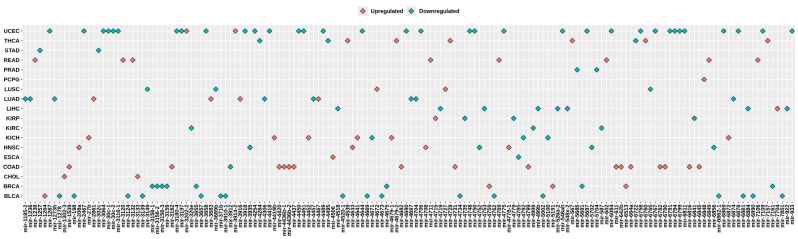
Cancer-specific differentially expressed miRNAs were identified in each cancer type

**Fig. 4:**
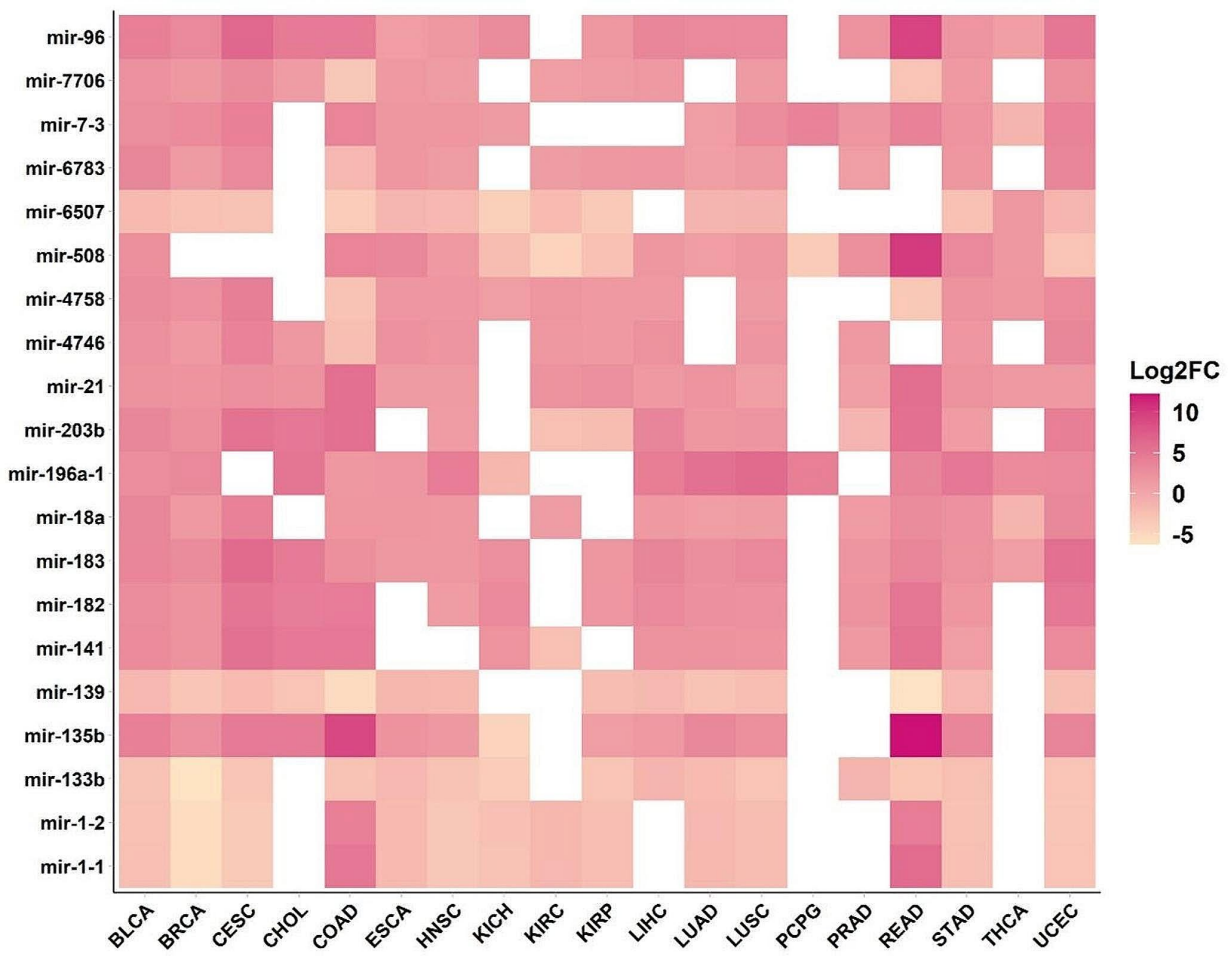
Differentially expressed top 20 common miRNAs in TCGA cancer types

Examining the differences between healthy and cancerous cells helps in understanding pathology and developing treatment strategies. A specific area of research interest is DEmiRs, which entails identifying miRNAs that display differential expression patterns in cancer (Hu et al. 2018). Further, researchers are anticipating that miRNAs will become a routine approach for developing personalized patient profiles, enabling more targeted therapeutic interventions (Condrat et al. 2020). Hence, the study focused on conducting a DEmiR analysis comparing normal vs. tumor samples across the cancer types. Normal samples are available only for 19 cancer types in the TCGA. Hence, we investigated the miRNA expression profile of these cancer types. The highest number of DEmiRs were identified in Uterine Corpus Endometrial Carcinoma (UCEC: 709 upregulated & 73 downregulated) followed by Bladder urothelial carcinoma (BLCA: 661 upregulated & 33 downregulated), Lung squamous cell carcinoma (LUSC: 553 upregulated & 62 downregulated), and Stomach adenocarcinoma (STAD: 441 upregulated & 36 downregulated). Volcano plot of DEmiRs in 19 cancer types is provided in Fig. 2. After conducting a Venn analysis, we have identified miRNAs that are expressed uniquely and those that are expressed commonly. The list of miRNAs with unique expression (upregulated or downregulated compared to their respective normal) is given in Fig. 3. The highest number of uniquely expressed miRNAs were noticed in UCEC (32 upregulated and 2 downregulated). Whereas miR-6848 is the only miRNA uniquely downregulated in Pheochromocytoma and Paraganglioma (PCPG). We have also illustrated the top 20 DEmiRs common among 19 cancer types (Fig. 4). Interestingly, mir-96, mir-183, and mir-21 were found to be significantly upregulated in 17 cancer types. Additional details and a list of DEmiRs are provided in Supplementary File 1. To explore cancer specific dysregulated clustered miRNAs, we have mapped 481 miRNAs (belonging to 159 miRNA clusters) with DEmiR data. Members of two distinct clusters, mir-96 and mir-183, were found to be upregulated in 17 cancer types including Bladder urothelial carcinoma (BLCA), Breast invasive carcinoma (BRCA), Cervical and endocervical cancers (CESC), Cholangiocarcinoma (CHOL), Colon adenocarcinoma (COAD), Esophageal carcinoma (ESCA), Head and neck squamous cell carcinoma (HNSC), Kidney chromophobe (KICH), Kidney renal papillary cell carcinoma (KIRP), Liver hepatocellular carcinoma (LIHC), Lung adenocarcinoma (LUAD), Lung squamous cell carcinoma (LUSC), Prostate adenocarcinoma (PRAD), Rectum adenocarcinoma (READ), Stomach adenocarcinoma (STAD), Thyroid carcinoma (THCA), and Uterine corpus endometrial carcinoma (UCEC). The mir-21 is also upregulated in 17 cancers, except KICH and PCPG. Further, mir-133b was found to be downregulated in 15 types of TCGA cancers (BLCA, BRCA, CESC, COAD, ESCA, HNSC, KICH, KIRP, LIHC, LUAD, LUSC, PRAD, READ, STAD, and UCEC). Four clusters, mir-214, mir-1912, mir-29a, and mir-550b-1 were identified as specifically activated in ESCA, KIRP, READ, and THCA respectively. The members of miRNA clusters originating from the X chromosome, mir-514a-3, mir-508, mir-514a-1, mir-509-3, and mir-514a-2 were observed to be entirely downregulated in KICH, Kidney renal clear cell carcinoma (KIRC), KIRP, PCPG, and UCEC. Similarly, this study has generated a substantial amount of information on cancer-specific activated and silenced clustered miRNAs. The specifics regarding differentially expressed clustered miRNAs can be found in Supplementary File 2.

The differential expression profile of miRNAs indicates their essential role in cancer pathogenesis. While alterations in commonly and uniquely expressed miRNAs may not always lead to significant biological effects, this information can be merged with other biological data in a high-throughput manner to construct a landscape of the disease targets, identify biomarkers for screening and monitoring strategies (Hanna et al. 2019). In the field of pharmaceutical and clinical research, these DEmiRs can serve as potential biomarkers, therapeutic targets, prognosis, and diagnosis (Shah and Shah 2020). Further, differentially expressed miRNAs hold significant promise as non-invasive biomarkers for multiple cancers due to their rapid obtainability, minimal risk, and stability in body fluids (Shademan et al. 2023).

### Mapping of methylation sites on clustered miRNAs

**Fig. 5:**
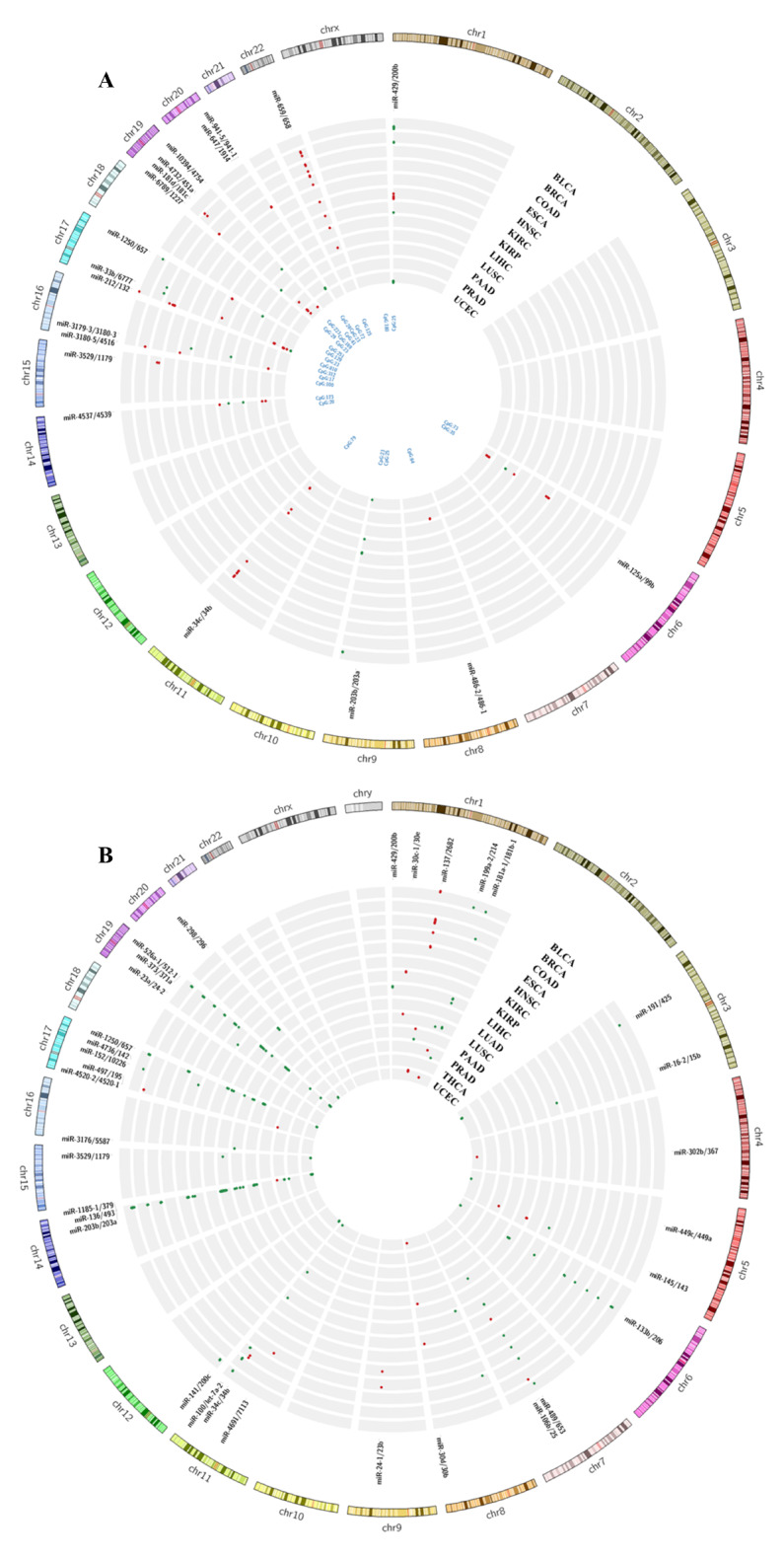
(**A**). Status of DM probes located upstream CpG islands of clustered miRNAs (**B**). Status of internal DM probes located inside the clustered miRNAs regions (Green: Hypomethylated; Red: Hypermethylated)

Many genes, including some tumor suppressors, have their promoter regions located within the CpG islands, which can be methylated in cancer; however, those under normal conditions are usually not methylated (Wajed et al. 2001). Further, many of these CpG islands become highly methylated and silences the gene expression (Deaton and Bird 2011). A similar process could silence the miRNAs with antitumor properties, that could potentially contribute to tumor development (Wang et al. 2017). In this computational study, we examine CpG islands located in the upstream region (~ 20 kb) of clustered precursor miRNA (pre-miRNA). Our goal was to test the hypothesis, that methylation of these CpG islands is linked to the regulation of clustered miRNA expression. A total of 74 miRNA clusters, comprising ~ 46% of the total clusters were recognized as having CpG islands located upstream of their promoter region (Supplementary File 3). After mapping methylation probes that were specific to cancer and their corresponding beta values onto these CpG islands, we determined that the CpG islands located at the promoters of 20 miRNA clusters across 12 cancer types showed distinct patterns of methylation (Fig. 5A). Interestingly, we observed that the probes mapped at CpG:125 in the promoter region of mir-200b/429 were hypermethylated in several cancer types, including BRCA, COAD, ESCA, KIRC, KIRP, LIHC, and LUSC. Also, we have identified two CpG islands, CpG:180 and CpG:25 located in the promoter of mir-200b/429. We found that CpG:180 was significantly hypermethylated only in KIRC, while all other probes mapped at CpG:180 and CpG:25 were significantly hypomethylated in BLCA, BRCA, KIRP, LIHC, LUSC, and UCEC. Based on our comparative analysis, we have found that certain miRNA clusters that exhibit a methylation pattern specific to a particular type of cancer and are distinct from other cancers. Specifically, we observed CpG:100 to be significantly hypermethylated in COAD, while the CpG:169 probe was found to be hypomethylated in LUSC. Further, various other cancer specific and common information on methylenation, CpG islands, probes, and clustered miRNAs is provided in Supplementary File 4.

### Case study: upstream CpG island regulates mir-200b/429 across pan-cancer

**Fig. 6:**
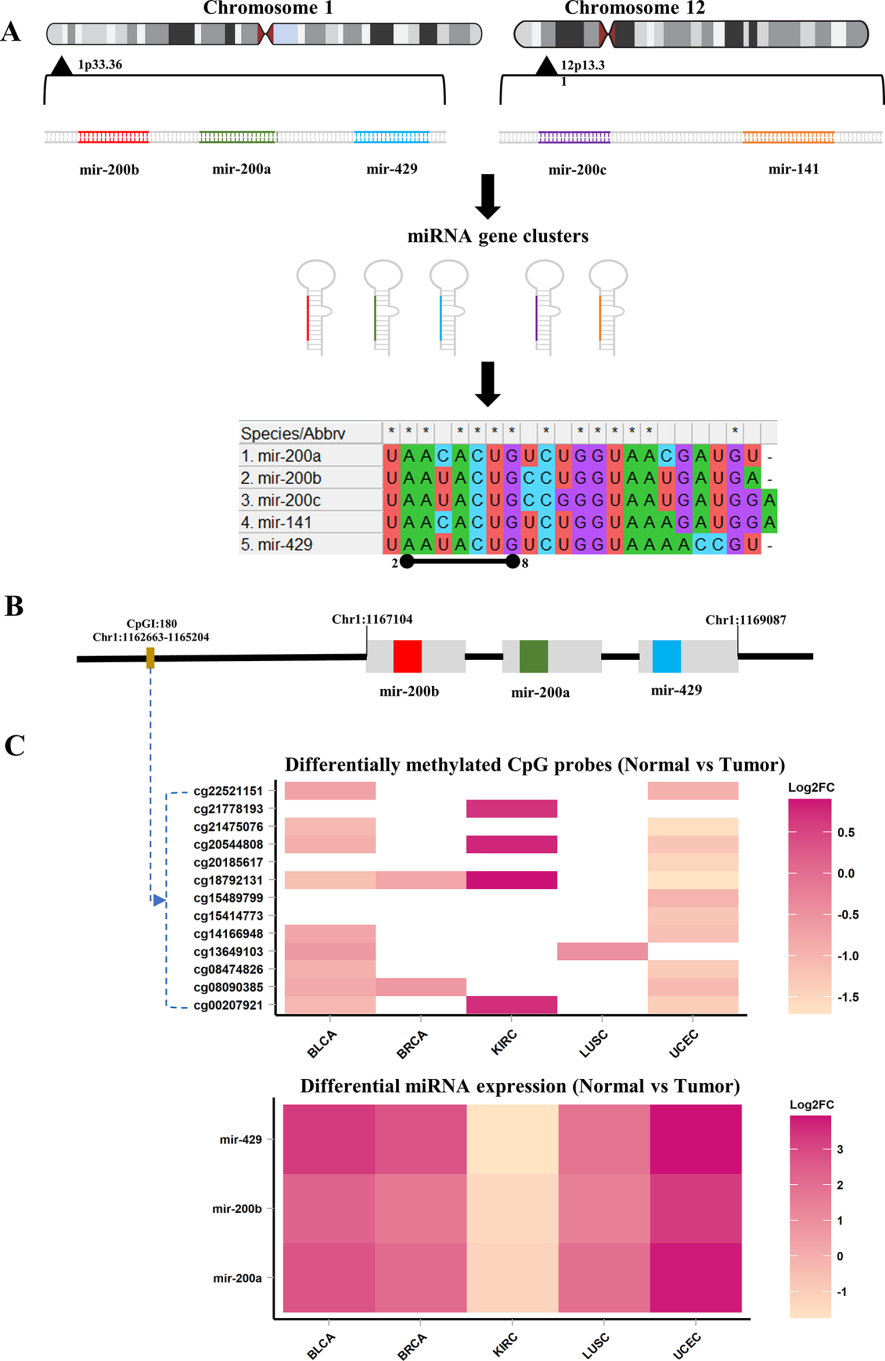
Integrative multi-omics analysis to understand the regulation of miRNA cluster mir-200b/429 by promoter CpG island (**A**). miR-200 family consists of two gene clusters: one on chromosome 1 (1p33.36) with miR-200b, miR-200a, and miR-429, and another on chromosome 12 (12p13.31) with miR-200c and miR-141 (**B**). CpG island (CpGI:180) is located approximately 4.44 kb from the miR-200b/429 cluster in the promoter region. (**C**). The expression of the miR-200b/429 cluster members is observed to be strictly controlled by the methylation status of CpGI:180

The mir-200b/429 cluster comprises three members, namely mir-200b, mir-200a, and mir-429, which are all part of the mir-200 family. Figure 6A provides a detailed overview of the mir-200 family, which arises from two gene clusters. The first cluster, located on chromosome 1 (1p33.36), contains miR-200b, miR-200a, and miR-429, while the second cluster, located on chromosome 2 (12p13.31), contains miR-200c and miR-141. Remarkably, an upstream CpG island (CpG:180) lies approximately ~ 4.44 kb away from this cluster, suggesting its potential significant involvement in regulating the entire cluster panel (Fig. 6B). To further emphasize and establish the significance of methylation patterns in the regulation of clustered miRNAs, we have examined and correlated promoter methylation with the expression of mir-200b/429 across various cancer types. We found a robust inverse correlation between upstream methylation at CpG:180 and the expression of cluster members in BLCA, BRCA, KIRC, LUSC, and UCEC (Fig. 6C). The Kaplan–Meier survival analysis revealed elevated expression of cluster members in BLCA and LUSC in association with patient mortality, indicating a positive correlation for methylation-driven mir-200b/429. Conversely, expression of this cluster in KIRC was associated with extended survival when expression was higher, while reduced expression indicated diminished survival probability (Supplementary Fig. 1).

Additionally, 362 potential target genes for mir-200b/429 have been obtained from the miRTabase. Upon integrating cancer-specific tumor suppressor and oncogene datasets from the TSGene and Oncogene databases, respectively. Interestingly, we determined that the mir-200b/429 targeted five tumor suppressors (*YAP1*, *THRB*, *RASSF8*, *DLC1*, *ABI2*) were downregulated in BLCA, BRCA, KIRC, LUSC, and UCEC. On the other hand, in KIRC, *LHX1* and *MYC* were recognized as oncogenes exhibiting upregulation, a trend inversely correlated with their regulator mir-200b/429. Moreover, the predictive potential of mir-200b/429 targeting various tumor suppressors and oncogenes is outlined in the Supplementary File 5, offering avenues for further exploration in diagnostic and prognostic applications. Various reports also corroborate the regulation of mir-200b/429 by promoter methylation in bladder cancer (Wiklund et al. 2011), breast cancer (Wee et al. 2012), and gastric cancer (Kurashige et al. 2015). Collectively, it can be confidently stated that the expression of the mir-200b/429 cluster is regulated by upstream promoter methylation. This case study demonstrates the latest functionalities of the CmirC aimed supporting integrated analysis.

### Internal regulators for clustered miRNAs

The diverse regulations of miRNAs play a crucial role in meeting the intricate demands of biological functions and aid in comprehending the complex functional and regulatory mechanisms of miRNAs at a network level (Liu et al. 2014). Clustered miRNAs can be classified into two types: homo-clusters, which contain miRNAs of the same family, and hetero-clusters, which contain miRNAs of different families (Wang et al. 2011). These two types of miRNA clusters can exhibit distinct behaviours of regulatory coordination in the gene regulatory network. Homo-clusters display direct regulatory coordination and are typically involved in biological processes of emergency situations, while hetero-clusters show indirect regulatory coordination and tend to participate in more complex biological processes. In addition, miRNAs belonging to the same cluster may demonstrate varying expression patterns. For example, the mir-379/656 (hereafter named as C14MC) miRNA cluster, the second largest known, has been associated with diverse developmental pathways and has also been implicated in conditions such as neurogenesis (Rago et al. 2014), neovascularization (Welten et al. 2014), and metabolic transition during birth (Labialle et al. 2014). It has also been reported that individual miRNAs from this cluster have been reported to be deregulated in leukemias (Olaru et al. 2011) and esophageal squamous cell carcinoma (Zhang et al. 2010). Additionally, specific key miRNAs (mir-134 and mir-485-5p) from C14MC have been shown to be capable of reducing glioblastoma tumorigenicity and can serve as potential future therapeutic markers (Nayak et al. 2018). Understanding the diverse expression patterns of miRNAs from the same cluster during carcinogenesis is crucial that is still in its early stages. So, by utilizing available data on CNV and DNA methylation, we tried to explore the impact of these regulators on clustered miRNA partial regulation and establish primary information that will show paths to future miRNA cluster-based research.

### CNV and clustered miRNAs partial co-localization

The fact that internal regulators can independently control clustered miRNAs, leading to diverse expression patterns among individual members, is well-established. Our study aimed to examine if CNVs have a partial effect on clustered miRNAs. We accomplished this by intersecting the coordinates of all significant aberrations identified across various types of cancer in TCGA with those of the clustered miRNAs. We identified 10 miRNA clusters (mir-6803/6804, mir-30c-1/30e, mir-6834/6873, mir-616/6758, mir-1185-1/379, mir-1-2/133a-1, mir-365a/193b, mir-133b/206, mir-100/let-7a-2, mir-152/10,226) that are partially colocalized with aberrant RCNV regions in BRCA, KIPAN, LGG, LIHC, LUAD, LUSC, SKCM, STAD, UCEC. We did not find any significant correlation between partially affected cluster member and its expression patterns. Further partially affected miRNA clusters and their details are provided in Supplementary File 6.

### Internal methylation in clusters

Epigenomic research has predominantly concentrated on abnormal DNA hyper and hypomethylation of specific gene sites located at promoters, enhancers, and gene bodies that contribute to tumor progression and cancer formation (Nishiyama and Nakanishi 2021). DNA hypermethylation affects gene expression in CpG rich promoter regions. These aberrations can potentially serve as biomarkers for various diseases. To explore the methylation trend inside the miRNA clusters, we have performed intersect analysis using the coordinates of miRNA clusters, CpG islands, CpG sites and their respective probes. We have identified that 32 cluster regions are entirely reside within the CpG islands region. All the details and locations of these CpG islands are provided in Supplementary File 7. Additionally, we have identified 34 clusters that have differentially methylated CpG sites located inside the gene body across 14 cancer types (Supplementary File 8). A comprehensive Circos illustration depicting the methylation status of all clustered miRNAs is presented in Fig. 5B. Notably, the largest human miRNA cluster mir-512-1/519a-2 (C19MC), located on chromosome 19 exhibits substantial internal CpG methylation across diverse cancer types, while all CpG sites appear to be hypomethylated. Likewise, we have detected CpG sites within mir-133b/206, mir-100/let-7a-2, and mir-1250/657 and observed that all these sites exhibited hypomethylation. On the other hand, mir-136/493 has the sites hypomethylated except in PRAD. Similarly, mir-137/2628 has all the sites hypermethylated except in PAAD. The study has uncovered several patterns that require further exploration to gain a better understanding of their role in regulating members of clustered miRNAs in a cancer-specific manner.

### Updated database statistics,** content and features**

**Fig. 7:**
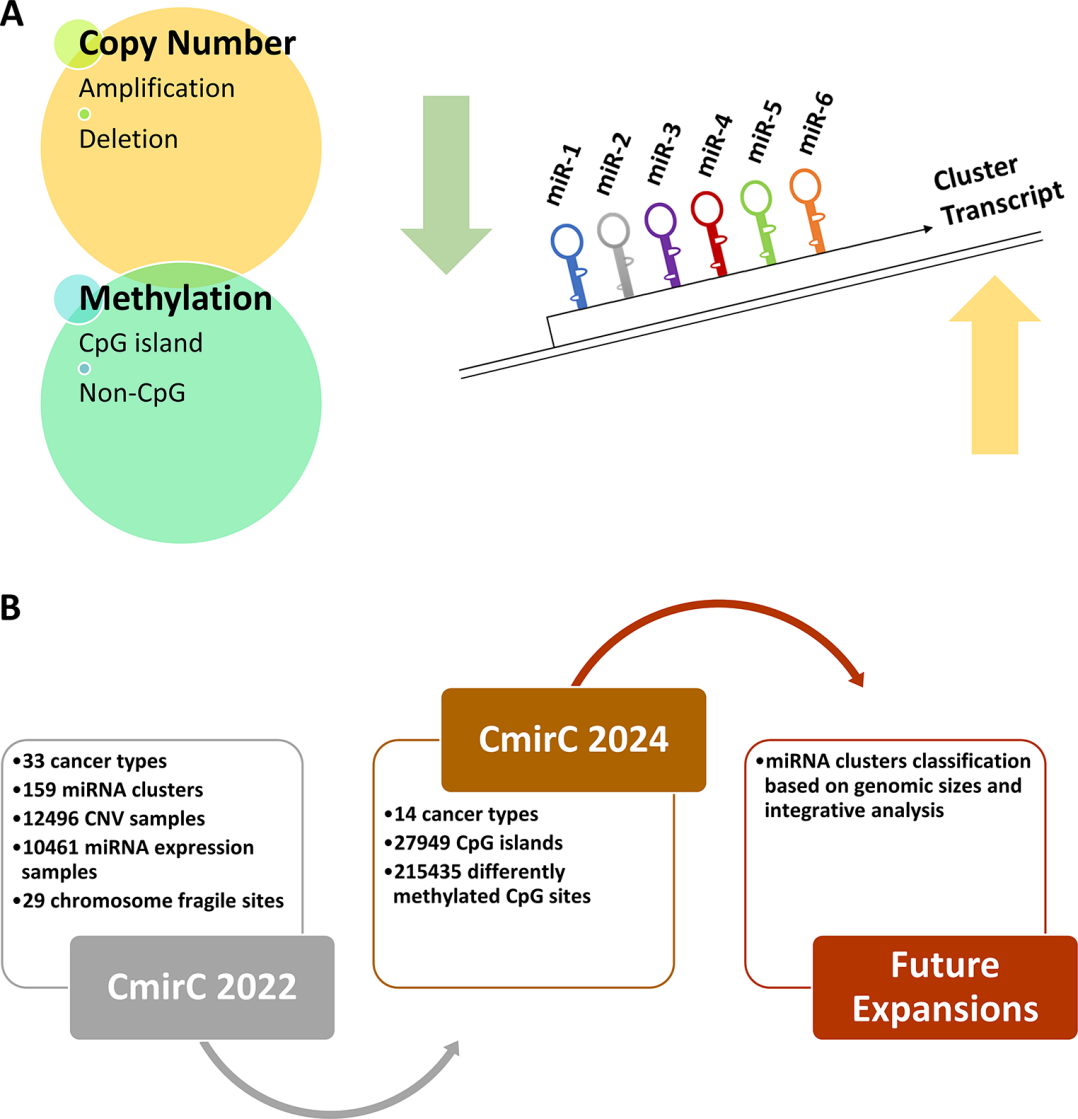
(**A**). Schematic representation of major genetic and epigenetic factors influence the clustered miRNAs. (**B**). Highlight of the additional data integration and future prospective of web-portal

The enhancements and new information included in the *CmirC* are outlined in Table 2. The *CmirC* is a pioneering upgraded online resource that provides multi-omics datasets related to clustered miRNAs in human cancers. The latest version of the *CmirC* has enhanced 14 cancer types with 27,949 CpG islands and over 215,435 DM probes. We have redesigned our genome browser to facilitate easy access to information about CpG islands and differentially methylated sites surrounding clustered miRNAs in TCGA cancers. Now, users can utilize methylation track at genome browsers to explore and compare methylation among several cancer types, with or without different CNV regions for a given clustered miRNA. As illustrated in Fig. 7A, differential expression of clustered miRNAs due to CNVs and DNA methylation can now be explored more efficiently with this portal. Moreover, it is now effortless to visualize the distribution of differentially methylated sites or regions across chromosomes and their association with miRNAs through the updated *CmirC* portal. With the updated functionality, users can easily navigate and visualize multiple parallel tracks of annotated features in a cumulative and seamless manner. The portal provides users with expression information, making it easy and convenient to perform correlation analysis between methylation and miRNA expression. Using the highly flexible and customizable option of JBrowse, users can easily upload miRNA, gene, methylation or CNV datasets, analyze, and download the reports. By hyperlinking individual members of clustered miRNAs to the UALCAN repository, *CmirC* enables users to draw Kaplan-Meier curves based on TCGA-miRNA expression datasets, provides more insights for cancer diagnosis and treatment guidance. Separate pages are provided to access general information about the corresponding miRNA, including the precursor mapping, mature miRNA sequence, and annotated genomic loci. Now each miRNA entry is linked to other third-party databases such as literature, cancer associated miRNA databases, miRNA expression database and most importantly miRNA target databases. The miRNA page provides users with comprehensive information about each miRNA, including details about the precursor mapping, mature miRNA sequence, and annotated genomic loci. These resources allow users to better usability and access to a wealth of information and gain a better understanding of the clustered miRNA and its potential functions. These enhancements in the *CmirC* can promote this web portal as a popular online resource for clustered miRNAs and cancer research. Figure 7B outlines the purpose of the upgrade and emphasizes the major improvements made to *CmirC*.

**Table 2:** A quick comparison between *CmirC* releases

Features	CmirC 2022	CmirC 2024
Last update	June 2022	June 2024
Known miRNA entry	miRBase v22	miRBase v22
Support species	*Homo sapiens*	*Homo sapiens*
Number of cancers	33 Cancers	33 Cancers
Methylation data from cancers	Not available	14 Cancers
No. of CNV samples	12,496	12,496
No. of miRNA expression samples	10,461	10,461
No. of methylation samples	Not available	9639
Total number of methylation sites	Not available	215,435
Total number of CpG islands	Not available	27,949
Number of miRNA clusters	159	159
Survival analysis	No	Yes
CpG Islands information	No	Yes
Link to literature databases	No	Yes
Link to miR-cancer databases	No	Yes

## Summary and conclusion

At present, research on the regulation of miRNA clusters during tumor development is still in its early stages. Additionally, there is a lack of comprehensive resources that offer information on both the genetic or epigenetic changes and functional regulation of clustered miRNAs in relation to carcinogenesis. To address this gap, we introduced *CmirC* in 2022, a web portal that offers information on 481 clustered miRNAs co-localized with CNV regions and their expression profiles across 35 cancer types. Here, the *CmirC* (2024) equipped with a broader range of CpG methylation datasets to support various research areas, including the design of biomarker panels, comparison of different cancer types, diagnosis, prognosis, therapy studies, and identification of potential epigenetic biomarkers. The *CmirC* is a comprehensive database of clustered miRNAs composed for integrative multi-omics analysis using the TCGA cancer datasets. The *CmirC* web portal enables quick and easy steps for experimental biologists to perform various computational analyses on diverse cancer types. Using *CmirC*, one can explore information on clustered miRNAs, miRNA expression, CNVs, DNA methylation, and perform *in silico* analysis to identify the answer to specific scientific questions. Studying the interplay between overlapping and non-overlapping CNV and methylation elements across clustered miRNAs can shed light on the activation mechanisms of various regulatory pathways in different cancer types. This analysis is also invaluable for deciphering key aspects of tumor heterogeneity. Given that clustered miRNAs are known to regulate gene expression in a tissue-specific manner, identifying survival-associated cluster members could pave the way for future cancer therapeutics. Moreover, targeting miRNA expression in a tissue-specific manner lays the foundation for cluster-based cancer treatment strategies. Continuously updating the database ensures its reliability as a vital platform for cancer researchers and the scientific community at large.

### Limitations and future perspectives

Differential expression of clustered miRNAs can alter the expression of oncogenes and tumor suppressor genes. Genetic and epigenetic factors can alter the expression of clustered miRNAs. An integrated genetic and epigenetic data analysis and accurate data interpretation can provide better understanding of miRNA-mediated regulatory mechanisms in cancer. Integrating multi-omics data, such as copy number variations and DNA methylation, poses substantial technical challenges due to differences in data formats, quality, and sources. Sophisticated computational approaches and robust algorithms are required to ensure accurate and meaningful analysis, which is resource-intensive. Maintaining and updating bioinformatics resource for clustered miRNA studies and ensure accuracy is an ongoing challenge. Regular updates demand continuous monitoring and validation to prevent data discrepancies and ensure reliability. Experimental validation of high throughput data analysis findings in global populations remains a critical bottleneck. Wet lab validation is necessary to translate computational insights into therapeutic applications. The heterogeneity among different cancer types adds another layer of complexity, as each type can exhibit unique miRNA expression profiles and regulatory mechanisms. Maintaining performance and scalability as the database grows requires infrastructure investment and manpower. Addressing these challenges is crucial for advancing our understanding of miRNA-mediated regulation in cancer and leveraging this knowledge for diagnostic and therapeutic innovations.

### Electronic supplementary material

Below is the link to the electronic supplementary material.

Supplementary Material 1

Supplementary Material 2

Supplementary Material 3

Supplementary Material 4

Supplementary Material 5

Supplementary Material 6

Supplementary Material 7

Supplementary Material 8

Supplementary Material 9

## Data Availability

All data sets analyzed during this study is freely available at CmirC web-portal (http://slsdb.manipal.edu/cmirclust/).
