# Supplementary material for: CmirC update 2024: a multi-omics database for clustered miRNAs
Source: Funct Integr Genomics. 2024 Aug 1;24(4):133. doi: 10.1007/s10142-024-01410-2 (PMC11291601; doi:10.1007/s10142-024-01410-2)
Supplement: Supplementary file 1 — Supplementary Material 1 [file 10142_2024_1410_MOESM1_ESM.docx]

**CmirC update 2024: A multi-omics database for clustered miRNAs**

Akshay Pramod Ware^1,2^, Kapaettu Satyamoorthy^3^, and Bobby Paul^1*^

^1^Department of Bioinformatics, Manipal School of Life Sciences, Manipal Academy of Higher Education, Manipal – 576104, Karnataka, India.

^2^Institute of Cardiovascular Regeneration, Johann Wolfgang Goethe University, Theodor-Stern-Kai 7, Frankfurt Am Main – 60590, Germany.

^3^SDM College of Medical Sciences and Hospital, Shri Dharmasthala Manjunatheshwara (SDM) University, Manjushree Nagar, Sattur, Dharwad, Karnataka, 580009, India.

***Correspondence to:** bobby.paul@manipal.edu

**Supplementary Data**

**
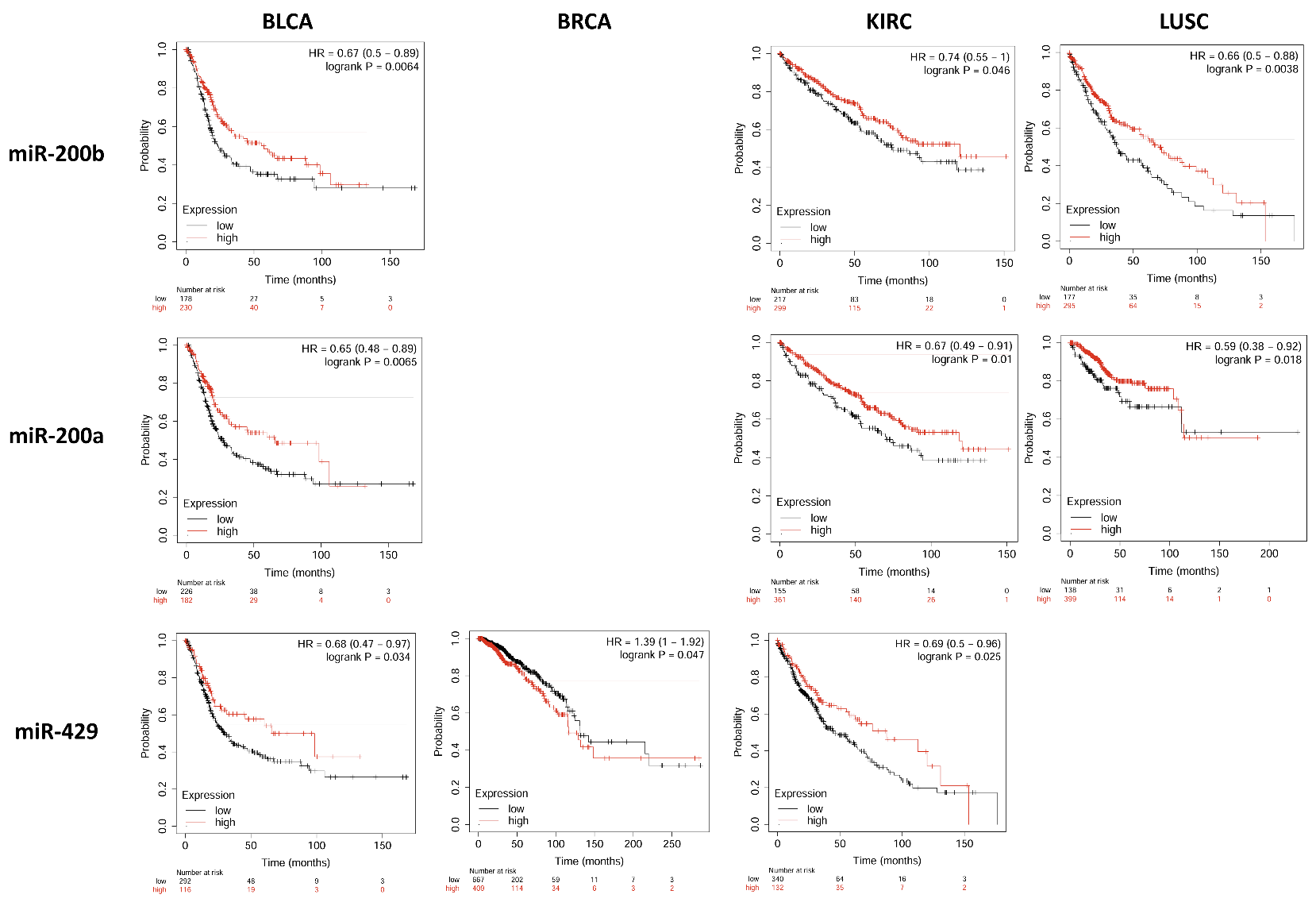
**

**Supplementary Figure 1.** The Kaplan–Meier survival curves show overall survival outcomes of mir-200b/42 miRNA cluster candidates.

**Supplementary Table 1.** List of total TCGA cancer samples and their available datasets utilized in the study.

| Abbreviation | Cancer Type | No. of CNV samples | miRNA Expression | |
| --- | --- | --- | --- | --- |
|  |  |  | **Healthy** | **Cancer** |
| TCGA-ACC | Adrenocortical carcinoma | 90 | NA | 80 |
| TCGA-BLCA | Bladder urothelial carcinoma | 410 | 19 | 417 |
| TCGA-BRCA | Breast invasive carcinoma | 1089 | 104 | 1096 |
| TCGA-CESC | Cervical squamous cell carcinoma and endocervical adenocarcinoma | 295 | 3 | 307 |
| TCGA-CHOL | Cholangiocarcinoma | 36 | 9 | 36 |
| TCGA-COAD | Colon adenocarcinoma | 451 | 8 | 455 |
| TCGA-COADREAD | Colorectal adenocarcinoma | 616 | NA | NA |
| TCGA-DLBC | Lymphoid Neoplasm Diffuse Large B-cell Lymphoma | 48 | NA | 47 |
| TCGA-ESCA | Esophageal carcinoma | 184 | 13 | 186 |
| TCGA-GBM | Glioblastoma multiforme | 577 | NA | NA |
| TCGA-GBMLGG | Glioma | 1090 | NA | NA |
| TCGA-HNSC | Head and Neck squamous cell carcinoma | 522 | 44 | 523 |
| TCGA-KICH | Kidney Chromophobe | 66 | 25 | 66 |
| TCGA-KIPAN | Pan-kidney cohort (KICH+KIRC+KIRP) | 883 | 130 | 901 |
| TCGA-KIRC | Kidney renal clear cell carcinoma | 528 | 71 | 544 |
| TCGA-KIRP | Kidney renal papillary cell carcinoma | 289 | 34 | 291 |
| TCGA-LGG | Brain Lower Grade Glioma | 513 | NA | 512 |
| TCGA-LIHC | Liver hepatocellular carcinoma | 370 | 50 | 372 |
| TCGA-LUAD | Lung adenocarcinoma | 516 | 46 | 519 |
| TCGA-LUSC | Lung squamous cell carcinoma | 501 | 45 | 478 |
| TCGA-MESO | Mesothelioma | 87 | NA | 87 |
| TCGA-OV | Ovarian serous cystadenocarcinoma | 586 | NA | 491 |
| TCGA-PAAD | Pancreatic adenocarcinoma | 184 | 4 | 178 |
| TCGA-PCPG | Pheochromocytoma and Paraganglioma | 175 | 3 | 179 |
| TCGA-PRAD | Prostate adenocarcinoma | 184 | 52 | 498 |
| TCGA-READ | Rectum adenocarcinoma | 165 | 3 | 161 |
| TCGA-SARC | Sarcoma | 257 | NA | 259 |
| TCGA-SKCM | Skin Cutaneous Melanoma | 469 | 3 | 97 |
| TCGA-STAD | Stomach adenocarcinoma | 442 | 45 | 446 |
| TCGA-TGCT | Testicular Germ Cell Tumors | 150 | NA | 150 |
| TCGA-THCA | Thyroid carcinoma | 499 | 59 | 506 |
| TCGA-THYM | Thymoma | 123 | 2 | 124 |
| TCGA-UCEC | Uterine Corpus Endometrial Carcinoma | 540 | 33 | 545 |
| TCGA-UCS | Uterine Carcinosarcoma | 56 | NA | 57 |
| TCGA-UVM | Uveal Melanoma | 80 | NA | 80 |

NA: Data is not available
